# Origin of Neuroblasts in the Avian Otic Placode and Their Distributions in the Acoustic and Vestibular Ganglia

**DOI:** 10.3390/biology12030453

**Published:** 2023-03-15

**Authors:** Matías Hidalgo-Sánchez, Antuca Callejas-Marín, Luis Puelles, Luis Sánchez-Guardado

**Affiliations:** 1 Department of Cell Biology, School of Science, University of Extremadura, E06071 Badajoz, Spain; acallejas@unex.es; 2 Department of Human Anatomy and Psychobiology, School of Medicine, University of Murcia, E30100 Murcia, Spain; puelles@um.es; 3 Murcian Institute of Biosanitary Research (IMIB-Arrixaca), E30100 Murcia, Spain

**Keywords:** chick/quail chimaeric embryos, sensory patch, vestibular ganglion, acoustic ganglion, neuroblasts

## Abstract

**Simple Summary:**

A key question in embryonic development is to determine the molecular and cellular mechanisms involved in the early specification and differentiation of neuroblast populations and the final disposition of mature neurons in the central and peripheral nervous system. These embryonic developmental events are governed by genetic factors intrinsic to each neuroblast subpopulation and by diffusible signals from the tissue environment. The inner ear is an attractive model for building a knowledge base in this field, as it is accessible to manipulation and undergoes dynamic self-organization, with significant morphogenetic changes. Using the chimeric chick/quail model, which provides stable cell markers of quail cells grafted into a chick tissue environment, long after transplantation experiments, our results clearly show that, in birds, there does not seem to be a strict segregation of acoustic and vestibular ganglion neurons in the otic placode. Segregation of both otic neuroblast populations occurs from the otic cup stage onwards, especially in the otic vesicle stage, once the clonal restriction compartments separating the distinct cell lineages have been defined. Further descriptive and experimental studies are needed to better characterize the gene expression mosaic of transcription factors and the signaling pathway governing neuronal fate acquisition in the early otic placode.

**Abstract:**

The inner ear is a complex three-dimensional sensorial structure with auditory and vestibular functions. This intricate sensory organ originates from the otic placode, which generates the sensory elements of the membranous labyrinth, as well as all the ganglionic neuronal precursors. How auditory and vestibular neurons establish their fate identities remains to be determined. Their topological origin in the incipient otic placode could provide positional information before they migrate, to later segregate in specific portions of the acoustic and vestibular ganglia. To address this question, transplants of small portions of the avian otic placode were performed according to our previous fate map study, using the quail/chick chimeric graft model. All grafts taking small areas of the neurogenic placodal domain contributed neuroblasts to both acoustic and vestibular ganglia. A differential distribution of otic neurons in the anterior and posterior lobes of the vestibular ganglion, as well as in the proximal, intermediate, and distal portions of the acoustic ganglion, was found. Our results clearly show that, in birds, there does not seem to be a strict segregation of acoustic and vestibular neurons in the incipient otic placode.

## 1. Introduction

The acquisition of specific fate assignment and the subsequent cell differentiation, clonal expansion, and later segregation in a spatial-temporal multiple step process are key events during embryonic development. Due to their dynamic self-organization and their accessibility for manipulation, developing vertebrate inner ears have been exceptional models for understanding these developmental processes, creating an excellent knowledge base. The vertebrate inner ear is a complex sensory organ, formed by fluid-filled cavities and chambers, and whose wall has specialized sensory and non-sensory organs involved in auditory and balance functions. The initial morphological evidence of the development of the incipient inner ear is the differentiation of the otic placodes, formed as transient thickened portions of the cephalic ectoderm, lying next to the developing hindbrain [1,2,3,4], extending from rhombomere 4 to the pro-rhombomere C levels in birds [5]. The otic placode invaginates to generate the otic cup, which then pinches off the cephalic ectoderm to create a simple pear-shaped structure, the otocyst or otic vesicle, from which the mature inner ear develops. All sensory elements of this intricate organ are composed of mechano-transducing hair cells that transform mechanical stimuli into electric signals and supporting cells that offer cellular and mechanical support to the hair cells, with both types of cells having an extremely specific cytoarchitectonic arrangement [6,7]. Neurons of the acoustic and vestibular ganglia connect the hair cells of the developing otic epithelium with their targets, the vestibular and auditory nuclei in the hindbrain, with a topographically well-defined pattern of connections [8,9,10,11]. Both hair cells and otic neuroblasts have a clonal relationship, these cells being derived from a population of common progenitor cells [12,13]. Dynamic spatial and temporal interactions between transcription factors and key signaling pathways govern the morphogenetic and specification events, as well as axon guidance mechanisms, that occur during the development of the vertebrate inner ear [14,15,16,17,18,19,20].

The generation of the otic neuroblasts is the first cell specification and differentiation event that takes place in the early invaginating otic placode [21,22,23]. After a step-by-step progressive specification in the incipient placodal epithelium [24], otic neuroblasts migrate across the basal lamina and proliferate in the subjacent mesenchyme, to finally differentiate into distinguishable auditory and vestibular ganglia [8,19,20,21,25,26,27,28,29,30,31]. The early *Fgf10* expression in the otic placode demarcates a proneural-sensory domain located in its most anterior-dorsal portion, where early proneural and neurogenic genes start to be expressed (*Ngn1*, *Delta1*, *and Hes5*), followed by *NeuroD* and *NeuroM*. These gene expression sequences govern the molecular mechanisms involved in the creation of a competent domain and in the subsequent specification, determination, and differentiation of early neuroblasts [24,26]. *Lfng* expression also confirmed the existence of this proneural-sensory domain, from which early otic neuroblasts delaminate [24]. In a complementary way, a non-neural-sensory domain was consequently defined in the most posterior-lateral portion of the otic placode, stained using *Lmx1* gene expression [32] and the location of the HNK1 epitope [24,33]. 

When development proceeds, delaminating neuroblasts, *Islet1/2*-positive, are detected exclusively in a narrow stripe located along the border of the *Fgf10*-labeling domain at the late otic cup stage [24,34]. At the otic vesicle stage, neurogenesis occurs in the anteroventral wall of the otocyst [21,22,35,36,37,38,39,40,41,42,43]. Thus, intense otic neurogenesis takes place in the presumptive areas of the utricular and saccular maculae [12,13,19]. Interestingly, several studies have shown that otic neurogenesis could also occur within the auditory organ [44,45,46,47]. As a final consequence, the axonal innervation of the developing sensory patches is carried out by these placodal-derived ganglionic neurons [48]. 

During embryonic development, diverse subpopulations of otic neuroblasts are generated from small areas of the developing otic epithelium, with differential molecular profiles. The segregation of auditory and vestibular neurons has been considered, with these two types of neurons probably obtaining their respective identities prior to migration from the otic epithelium. In mice, *GATA3* is expressed in auditory, but not in vestibular, neurons [49,50,51]. Thus, auditory and vestibular neuroblasts could originate from specific areas of the ventral otocyst epithelium, defined by the expression patterns of *GATA3* and *NeuroD* [52]. Using a *Ngn1-CreER^T2^* transgenic mouse line, it was reported that early *Neurog1*-expressing cells mostly give rise to the vestibular neurons, whereas the late *Neurog1*-expressing cells predominantly generate cochlear neurons [53]. A spatial relationship between subpopulations of otic neurons and developing sensory patches was also suggested, such that the specification of the vestibular neurons and the utricular macula are coupled, as are the auditory neurons and the saccular macula [34,41]. In addition, studies on chick development have shown that the precursors of auditory and vestibular cells are regionally segregated in the epithelium of the otic placode/cup, with vestibular neurons being generated in its anterior-lateral portion, while auditory neurons are originated in its posterior-medial region [54]. As a consequence, positional information with respect to nearby tissues, mediated by different signaling pathways (FGF, retinoic acid, and SHH, among others), could be responsible for the appropriated specification of both vestibular and auditory neurons at the otic placode stage. 

To better examine whether the origin-relative topological dissimilarities among different subpopulations of otic neuroblasts in the otic placode could be essential for early fate assignment and future differentiation and segregation in the mature vestibular and acoustic ganglia (VG and AG), an experimental study using the chick/quail chimeric graft method was performed. Considering our previous fate map of the avian otic placode [5] and earlier otic connection studies [48], we grafted small portions of the otic placode containing the presumptive territory of specific sensory patches, together with small portions of the nearby prospective non-sensory epithelium. Such mixed grafts were designated as “expanded sensory areas” [48]. It is worth noting that the set of transplants contained the neurogenic zone of the otic placode [24,33], except the grafts containing the presumptive territory of the posterior crista, located in the most posterior-ventral portion of the chick otic placode [48]. It is also interesting to highlight that each type of otic placode graft contained otic neuroblast precursors that generated otic neurons throughout the entire otic neurogenic period. The location of the grafted quail cells in the AG and VG was determined using QCPN- and QN-immunoreactions at stage HH38 (10 days of incubation), when every sensory patch was completely differentiated and the anatomical aspects of the ganglia could be clearly identified [55,56,57,58]. Our results clearly show that there was no segregation of the vestibular and acoustic neuroblasts according to the placodal topologic domain from which they delaminated. The possibility of lineage restriction compartments within the avian otic placode should be ruled out. These results could help to better understand the molecular and cellular mechanisms underlying the early specification and subsequent morphological segregation, neuronal differentiation, and axonal pathfinding processes of vestibular and auditory neurons.

## 2. Materials and Methods

### 2.1. Processing of the Tissue

Experiments were performed using White Leghorn chickens (*Gallus gallus*) and Japanese quail (*Coturnix coturnix japonica*). Fertilized eggs were incubated at 38 ± 1 °C for a maximum of 10 days (stage HH34). Experimental animals were fixed, and serial sections of the inner ear were obtained in a cryostat and stored at −80 °C until use [55,56,59].

### 2.2. Grafting Experiments

Quail–chick grafts were performed by replacing a specific portion of the chicken otic placode at the stage HH10 (10-somate stage [60]), long after the extensive cell movements that occur during the earlier stages of otic placode formation had finished [1], as confirmed in our previous fate map [5]. Unilateral homotopic and isochronic transplants were carried out based on our previous fate mapping study [5,48]. Six types of grafting experiment were performed, containing the expanded sensory area of the macula utriculi (type-1 graft; n = 9), of the macula sacculi (type-2 graft; n = 9), of the basilar papilla (type-3 graft; n = 8), of the macula lagena plus the macula neglecta (type-4 graft; n = 8), of the anterior crista plus the lateral crista (type-5 graft; n = 7), and of the posterior crista (type-6 graft; n = 6). Quail–chick chimeric grafts have been described by [61]. As it is well known that small portions of the otic epithelium contiguous to the developing sensory elements can generate neuroblasts, all transplants contained the presumptive territory of the sensory elements, plus small portions of the adjacent non-sensory epithelium, the so-called “extended sensory zones”. In all grafting experiments, QCPN labelling in the otic epithelium was analyzed to confirm that the entire sensory area under consideration was included in the graft. On the other hand, it is methodologically impossible to exclusively and completely transplant the presumptive territory of a sensory element without including some cells from the contiguous non-sensory territory. When the transplanted non-sensory territory was very extensive, these chimera embryos were discarded and were not taken into account in our study. To avoid cell contamination from the hindbrain and neural crest to the inner ear [62,63,64], the grafted territories exclusively contained small portions of the cephalic ectoderm. However, ectodermal grafts may contain part of the underlying mesenchyme, so that some quail cells may be observed in the periotic mesenchyme. After the grafting procedure, eggs were closed and incubated until they reached stage HH34 (10 days of incubation), when the inner ear presents a mature morphology and all sensory elements are innervated by neurons from the acoustic-vestibular ganglion [55,56,57,58,65,66,67,68]. 

### 2.3. Immunohistochemical Staining Procedure

To localize the cell body of quail transplanted cells in the chimeric embryos, QCPN (DSHB; 1/100) mAb antibodies were used, while QN (1/10, a kind gift from Dr. Tanaka; [69]) was used to visualize the neuronal processes. These antibodies were visualized using sheep anti-mouse (1/100; Jackson ImmunoResearch) and mouse-PAP (1/200; Jackson ImmunoResearch) antibodies. The QN and QCPN immunocytochemistry reactions were performed after an anti-endogenous peroxidase treatment with 0.25% KMNO4 solution in PBS, followed by a wash in 1% oxalic acid solution in PBS [70]. The staining procedure was carried out by incubating the sections with a solution containing 0.03% of 3,3′-diaminobenzidine, 0.6% nickel ammonium sulphate, and 0.005% H_2_O_2_ in PBS. 

## 3. Results

**Figure 1:**
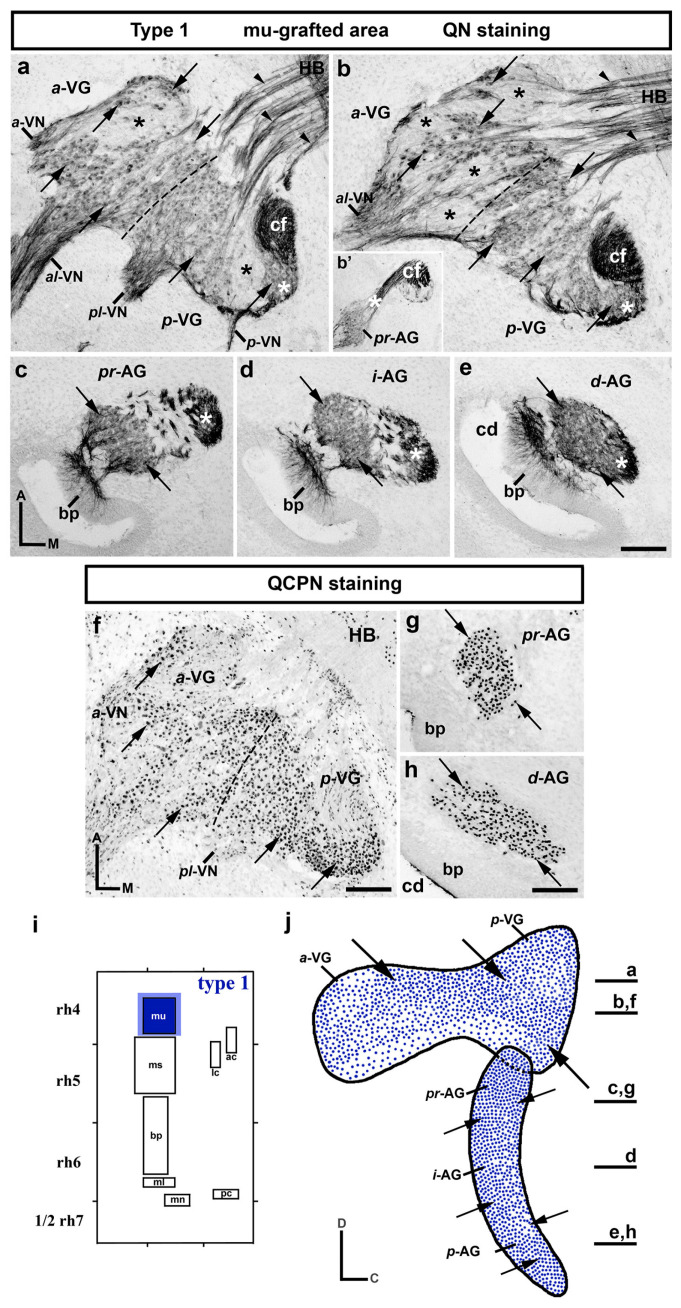
Graft-derived quail ganglionic neurons from the extended area of the macula utriculi (type-1 grafts; n = 9). (**a**–**h**) Serial horizontal sections stained with QN (**a**–**e**) and QCPN (**f**–**h**) antibodies through the vestibular (VG in (**a**,**b**,**f**)) and acoustic (AG in (**c**–**e**,**g**,**h**)) ganglia. (**a**,**b**,**f**) The dotted lines define the separation between the anterior and posterior lobes of the VG (*a*-VG and *p*-VG). The four nerves arising from the VG were also indicated in (**a**,**b**,**f**) (*a*-VN, *al*-VN, *pl*-VN, and *p*-VN). QN- and QCPN-stained grafted quail neurons were undoubtedly detected in the VG (*a*-VG and *p*-VG; QN, arrows in (**a**,**b**); QCPN, arrows in (**f**)). (**a**,**b**) The black asterisks indicate the areas of the VG devoid of mu-grated neurons. A higher density of grafted quail neurons was observed in the entire AG (*pr*-AG, *i*-AG, and *d*-AG; QN, arrows in (**c**–**e**); QCPN, arrows in (**g**,**h**)). (**a**–**e**) The white asterisks mark the QN-stained axonal fascicle from the basilar papilla (bp) and macula lagena, associated to the cochlear nerve fascicle (cf in (**b**,**b′**)). (**a**,**b**)The arrowheads point to grafted-neuron axons towards the hindbrain (HB in (**a**,**b**)). (**i**) Schematic representation of type-1 grafts performed at the 10-somite stage, taking a small portion of the chick otic containing the expanded sensory area of the macula utriculi (mu; dark and light blue areas). (**j**) Diagram showing a lateral view of the VG (dorsal) and AG (ventral), summarizing all these results in a lateral view of the VG and AG, in which the horizontal sections are indicated. The arrows point to the distribution of mu-grafted quail neurons. The afferent fibers of the basilar papilla were also observed (bp in (**c**–**e**); [48]). Orientation: A, anterior; C, caudal; D, dorsal; M, Medial. Additional abbreviations: cd, cochlear duct. Scale bar = 19 µm in (**e**) (**c**–**e**); 23 µm in (**a**,**b**,**f**); 17 µm in (**g**,**h**).

A relevant issue in vertebrate inner ear development is to determine the precise correlation between the origin of different subpopulations of otic neuroblasts and their later disposition in the acoustic and vestibular ganglia. To address this question, we performed homotopic chick/quail transplants of small portions of the otic placode at exactly the 10-somite stage (Hamburger Hamilton (HH) 10). These small grafts contained the presumptive territories of otic sensory areas and their contiguous non-sensory epithelia, according to our previous fate map study [5]. The resulting chimeric embryos were analyzed in horizontal sections at 8–10 days of incubation (HH32-36), when the anatomical aspects of the inner ear are completely established and the acoustic and vestibular ganglia are entirely segregated [55,56,57,58,68]. To visualize the grafted quail cells in the chick otic epithelium and in both ganglia, mAb QN and QCPN monoclonal antibodies were used. QN immunoreaction binds to some membrane molecules of quail nervous tissues, but not to chick tissues, with this method being an excellent marker for tracing neural processes in chick/quail chimeric embryos [48,69] (Figure 1a–e). QN staining helps to identify quail grafted neurons in the acoustic and vestibular ganglia from non-neuronal cells [71], and QCPN immunoreaction staining is useful to visualize the nuclei of quail transplanted cells in the developing central nervous system and inner ear [5,70,72]. QCPN staining was used to verify the location of the placodal grafted area in the otic epithelium and to confirm the location of graft-derived QN-stained neurons in the vestibular and acoustic ganglia (Figure 1f–h). 

Regarding the anatomical aspect of the vestibular and acoustic ganglia, studies of otic neuron segregation and axonal projections in both mammals and birds have considered the division of the vestibular ganglion into two portions, one superior portion and another inferior portion [10,54,73,74]. Judging from a preliminary anatomical study of the vestibular and acoustic ganglia in chicks using HuC/D immunoreactions, which labeled the postmitotic neuronal phenotypes [75], the vestibular ganglion can be considered to be divided into two portions (lobes), anterior (*a*-VG) and posterior (*p*-VG) (Figure 1a,b; Supplementary Figure S1), according to the anterior-to-posterior axis of the hindbrain. This anatomical consideration could be similar to that observed in the trigeminal ganglion, which is comprised of two portions: the ophthalmic lobe and the maxillomandibular lobe [62,76,77].

A detailed analysis of the VG using the Sox2 and 3A10 antibodies to label ganglionic cells [55,56,57,58,68,78,79], as well as the QN immunoreaction in chimeric embryos (Figure 1a-e), showed that two nerves arose from of each of its lobes. The most anterior nerve of the *a*-VG innervates the anterior and lateral cristae (*a*-VN in Figure 1a; Supplementary Figure S2), whereas its anterior-lateral nerve innervates the macula utriculi (*al*-VN in Figure 1a,b; Supplementary Figure S2). Considering the *p*-VG, its posterior-lateral nerve innervates the macula sacculi (*pl*-VN in Figure 1a; Supplementary Figure S2), whereas its most posterior nerve contains axons near the posterior crista and the macula neglecta (*p*-VN in Figure 1a; Supplementary Figure S2). The anterior-to-posterior disposition of these vestibular nerves fits in well with the topological distribution of these sensory elements in the chick otic placode [5]. 

Considering exclusively morphological evaluations, without taking into account tonotopic aspects, three subdivisions could be considered in the AG along the proximal-to-distal axis of the cochlear duct: proximal (*pr*-AG), intermediate (*i*-AG), and distal (*d*-AG) (Figure 1c–e; see also Supplementary Figure S1). The sensory elements located in the cochlear duct, the basilar papilla and the macula lagena, receive axons directly from the near AG. Axonal fascicles were detected in the medial-caudal and medial portion of the *i/d*-AG and *pr*-AG, respectively (white asterisks in Figure 1c–e; [80]. At least part of the fibers from the basilar papilla form a compact portion of the cochlea nerve fascicle when they go through the *p*-VG (cf in Figure 1a,b,b′; see also the white asterisk in Figure 1b′), whereas axons from the macula lagena cross the most caudal-peripherical portion of the *p*-VG (white asterisks in Figure 1a,b; see below).

### 3.1. Neuroblast Generation from the Maculae and Basilar Papilla Presumptive Domains of the Otic Placode

In chicks, the otic placode is constituted by three dorsoventral bands, from which all sensory and non-sensory elements develop. The maculae and basilar papilla originate from the arranged anterior-to-posterior domains, located between the presumptive domains of the endolymphatic system (dorsal) and those of the semicircular canals and their associated cristae (ventral) [5]. In this developmental context, the macula utriculi is topologically the most anterior macula (mu in Figure 1i). The type-1 chimeric grafts contained the expanded sensory area of the macula utriculi, i.e., the presumptive domain of the macula utriculi plus the most contiguous non-sensory epithelium [48]. When these chimeric embryos were analyzed in serial horizontal sections (Figure 1a–e), a high number of mu-grafted neurons were detected in both the anterior and posterior lobes of the VG (*a*-VG and *p*-VG; arrows in Figure 1a,b). An apparently higher number of grafted quail neurons were observed in the dorsal portions (Figure 1a,b) than in the most ventral portions. When the AG of the type-1 experimental embryos was examined, a high number of QN-stained grafted quail neurons were also clearly found in its proximal (*pr*-AG; arrows in Figure 1c), intermediate (*i*-AG; arrows in Figure 1d), and distal (*d*-AG; arrows in Figure 1e) portions. The results of the type-1 grafts were confirmed using the QCPN antibodies, which clearly identified the nuclei of the quail neurons (arrows in Figure 1f–h). Figure 1i corresponds to the schematic representation of type-1 grafts performed at the 10-somite stage, whereas Figure 1j summarizes the distribution of QN/QCPN-positive mu-grafted neurons in both ganglia of the type-1 experimental embryos.

**Figure 2:**
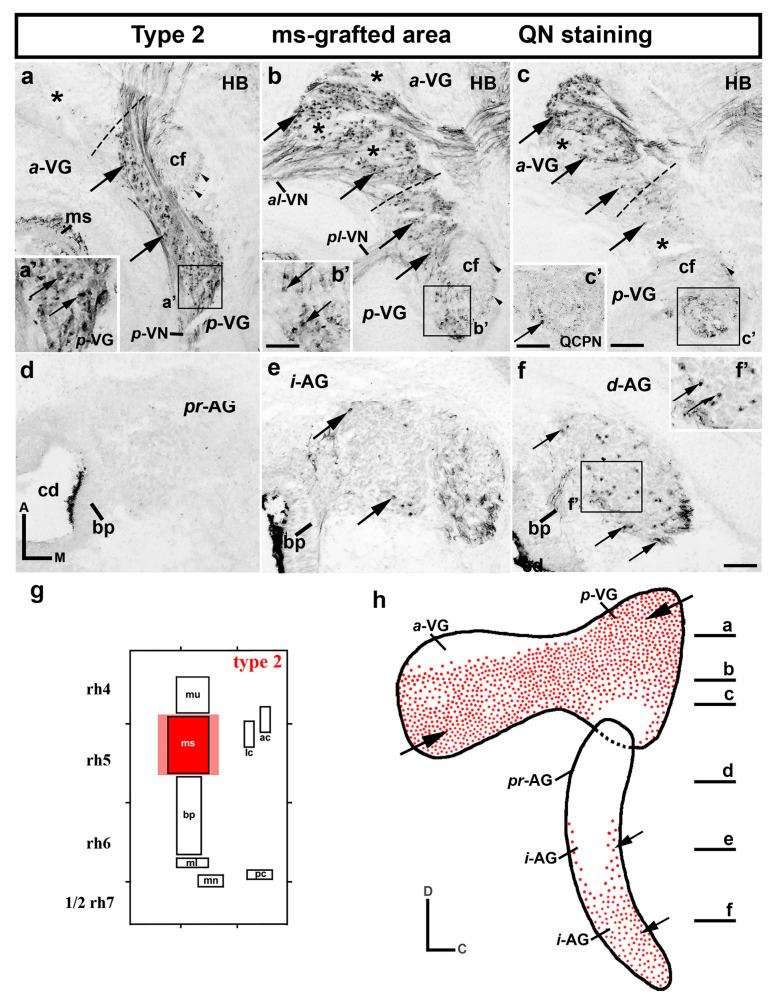
Graft-derived quail ganglionic neurons from the extended area of the macula sacculi (type-2 grafts; n = 9). (**a**–**f**) serial horizontal sections stained with QN antibodies through the vestibular (VG in (**a**–**c**)) and acoustic (AG in (**d**–**f**)) ganglia. (**a**–**c**) The dotted lines define the separation between the anterior and posterior lobes of the VG (*a*-VG and *p*-VG). QN-stained grafted quail neurons were detected in areas of the *a*-VG and *p*-VG (arrows in (**a**–**c**)), with other areas devoid of ms-grafted neurons (asterisks in (**a**–**c**)). In the AG, the *i*-AG and *d*-AG displayed a reduced number of QN-positive neurons (arrows in (**e**,**f**)), with the *pr*-AG without any QN-positive quail neurons (*pr*-AG in (**d**)). (**a**–**c**) The arrowheads point to axons in the cochlear nerve fascicle (cf). (**g**) Schematic representation of type-2 grafts performed at the 10-somite stage, considering the expanded sensory area of the macula sacculi (ms; dark and light red areas). (**h**) Diagram summarizing the results, in which horizontal sections are indicated. The arrows point to the most significant accumulations of ms-grafted quail neurons. Orientation: A, anterior; C, caudal; D, dorsal; M, Medial. Additional abbreviations: *al*-VN, anterolateral vestibular nerve; *ap*-VN, anteroposterior vestibular nerve; bp, basilar papilla; cd, cochlear duct; HB, hindbrain; ms, macula sacculi; *p*-VN, posterior vestibular nerve; *pl*-VN, posterolateral vestibular nerve. Scale bar = 11 µm in (**a′**,**b′**); 20 µm in (**a**,**b**,**c**); 14 µm in (**c′**); 12 µm in (**d**,**e**,**f**); 8,5 µm in (**f′**).

In the chick otic placode, the presumptive territory of the macula sacculi is located just caudal to the territory from which the utricular macula originates [5]. In the type-2 experiments, the small grafted portion of the otic placode contained the prospective macula sacculi area plus its contiguous non-sensory element (red areas in Figure 2g). When type-2 chimeric embryos were analyzed in serial horizontal sections (Figure 2a–f), mu-derived quail ganglionic neurons were also detected in both portions of the VG (*a*-VG and *p*-VG; arrows in Figure 2a–c). When the *p*-VG was analyzed, a high number of QN-positive neurons were observed in the dorsal horizontal sections (arrows in the *p*-VG in Figure 2a,a′,b,b′). The ventral half of the *p*-VG displayed a lower number of quail neurons (arrow in the *p*-VG in Figure 2c), with some areas devoid of them (lower black asterisk in Figure 2c). In the *a*-VG, horizontal sections through its most dorsal aspect showed that quail neurons were absent (asterisk in Figure 2a). In the adjacent ventral section (Figure 2b), the *a*-VG was occupied almost entirely by grafted neurons (arrows in the *a*-VG in Figure 2b), though they were missing in some areas (asterisks in Figure 2b). In the most ventral section through the *a*-VG (Figure 2c), quail neurons were detected mainly in its most rostral portion, its caudal half being occupied by a more reduced number of QN-stained neurons (Figure 2c). 

**Figure 3:**
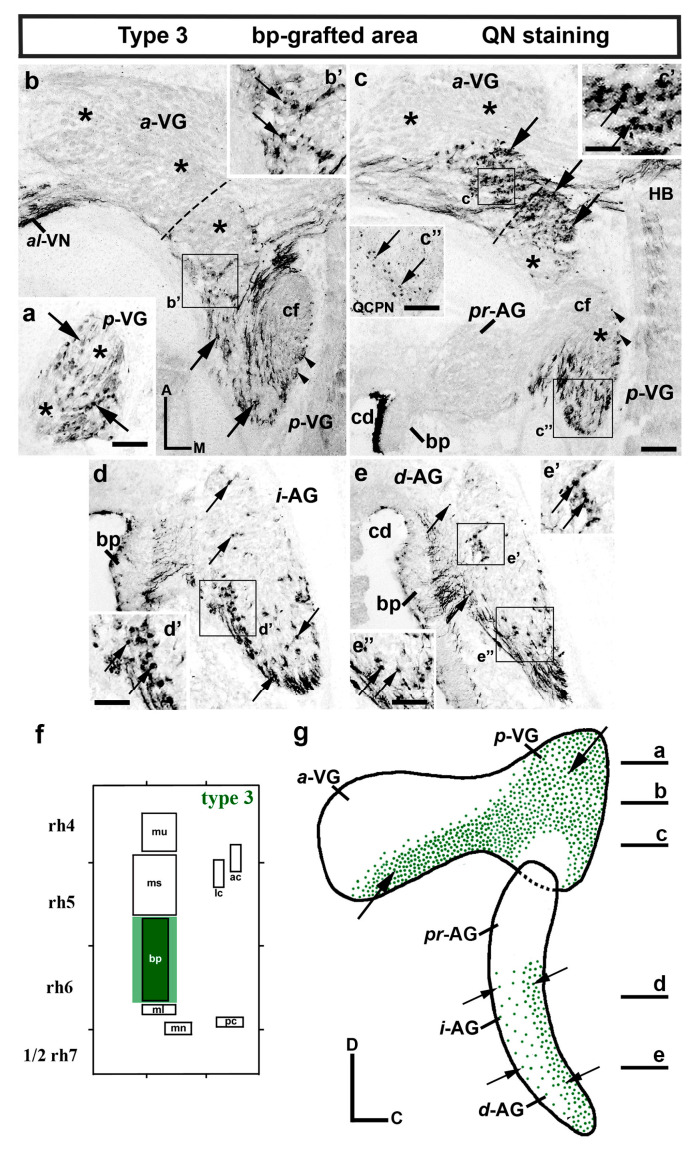
Graft-derived quail ganglionic neurons from the extended area of the basilar papilla (type-3 grafts; n = 8). (**a**–**e**) Horizontal sections stained with QN antibodies through the vestibular (VG in (**a**–**c″**)) and acoustic (AG in (**c**–**e′**)) ganglia. The dotted lines in b and c define the separation between the anterior and posterior lobes of the VG (*a*-VG and *p*-VG). QN-stained grafted quail neurons were detected in the vestibular ganglion (*a*-VG and *p*-VG; arrows in (**a**–**c″**)) and in the *i*-AG and *d*-AG (arrows in (**d**–**e′**)). The insert (**c″**) confirms the presence of QCPN-stained neurons in the *p*-VG. (**a**–**c**) The asterisks indicate the areas of the VG devoid of bp-grafted neurons. (**b**) The arrowheads point to axons in the cochlear fascicle (cf). (**f**) Schematic representation of type-3 grafts performed at the 10-somite stage, considering the expanded sensory area of the basilar papilla (bp; dark and light green areas). (**g**) Diagram summarizing these results, in which horizontal sections are indicated. The arrows point to the most significant accumulations of grafted quail neurons. Orientation: A, anterior; C, caudal; D, dorsal; M, Medial. Additional abbreviations: *al*-VN, anterolateral vestibular nerve; bp, basilar papilla; cd, cochlear duct; HB, hindbrain. Scale bar = 14 µm in (**a**); 18 µm in (**b**,**c**); 6.5 µm in (**b′**,**c′**); 12 µm in (**d**); 7 µm in (**d**′); 15 µm in (**e**); 10 µm in (**e′**,**e″**).

In the chick otic placode, the presumptive domain of the basilar papilla is located just caudal to the area from which the macula sacculi generates [5]. The type-3 chimeric embryos contain the expanded sensory area of the basilar papilla (green areas in Figure 3f). It is interesting to highlight that bp-grafted neurons were clearly observed in the VG (arrows in Figure 3a–c″). In the *p*-VG, a high number of QN-stained cells were distributed almost homogeneously in its most dorsal portion (arrows in Figure 3a), although quail-grafted neurons were missing in some areas (asterisks in Figure 3a). In ventral horizontal sections through the central aspect of the *p*-VG (Figure 3b), the quail-grafted neurons were located in the caudal-lateral portion (arrows in Figure 3b,b′), with the caudal-medial portion being largely filled by the cochlear fascicle (cf in Figure 3b). In a more ventral section through the *p*-VG (Figure 3c), grafted neurons were observed in the most anterior-medial part (arrows in the *p*-VG in Figure 3c) and in the most posterior-lateral periphery (arrows in Figure 3c″; QCPN staining). At this level, the rostral area was taken up by the *p*-VG quail neurons extending into the most caudal portion of the *a*-VG (arrow in the *a*-VG in Figure 3c; arrows in Figure 3c′), but not in its most rostral portion (asterisks in the *a*-VG in Figure 3c). The absence of QN-stained neurons in the *a*-VG was much more evident in the dorsal sections, where these kinds of neurons were not observed (asterisks in the *a*-VG in Figure 3b). Concerning the AG, its most proximal portion was completely devoid of bp-grafted quail neurons (*pr*-AG in Figure 3c). The QN-positive neurons observed were mainly located in the caudal half of the intermediate and distal portions (*i*-AG and *d*-AG; arrows in Figure 3d–e′). The results of the type-3 grafts were confirmed using QCPN antibodies (see Figure 3c″ for the *p*-VG). Figure 3g summarizes the distribution of QN/QCPN-positive neurons in the bp-grafted area (Figure 3f) in the VG and AG of the type-3 experimental embryos. 

**Figure 4:**
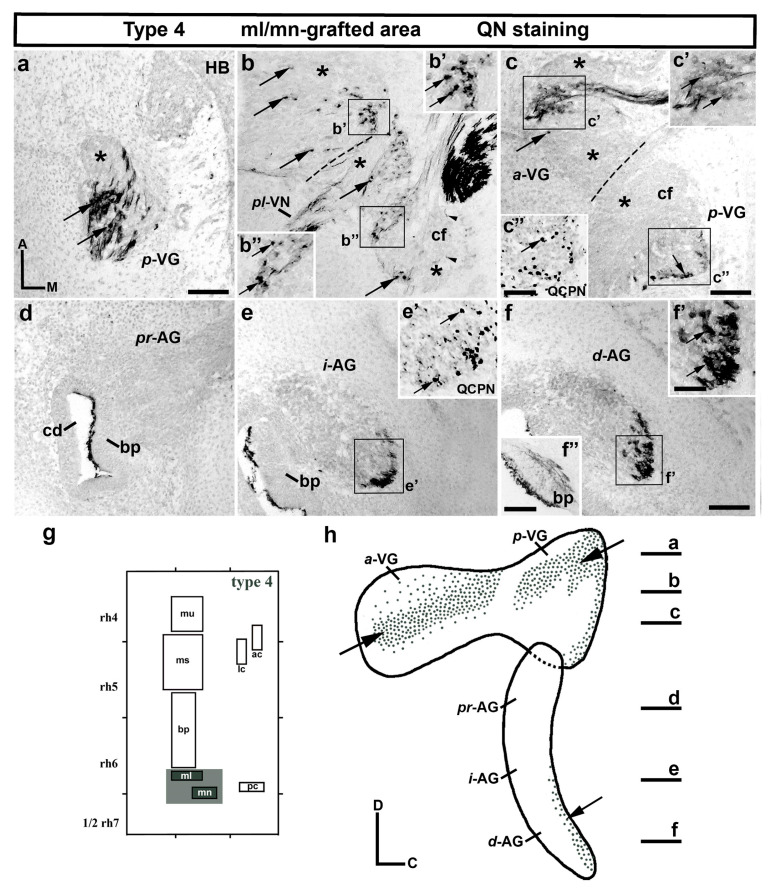
Graft-derived quail ganglionic neurons from the extended area of the macula lagena and macula neglecta (type-4 grafts; n = 8). (**a**–**f**) Horizontal sections through the vestibular (**a**–**c**) and acoustic (**d**–**f**) ganglia. QN-stained neurons were detected in the vestibular ganglion (*a*-VG and *p*-VG; arrows in (**a**–**c″**)). (**a**–**c**) The asterisks indicate the portions of the VG devoid of quail grafted neurons. In the AG, quail-grafted neurons were found in the periphery of the intermediate and distal subdivisions (*i*-AG and *d*-AG; arrows in (**e′**,**f′**)). Inserts (**c″**,**e′**) confirm the presence of QCPN-stained neurons in the *p*-VG and *i*-AG, respectively. Note that the proximal *pr*-AG was completely devoid of QN-positive neurons (**d**). The arrowheads in b point to a few axons in the cochlear fascicle (cf). (**g**) Schematic representation of type-4 grafts, considering the expanded sensory area of the macula lagena and macula neglecta (ml and mn; dark and light grey areas). (**h**) Diagram summarizing all these results, in which horizontal sections are indicated. The arrows point to the most significant accumulations of ml/mn-grafted neurons. Orientation: A, anterior; C, caudal; D, dorsal; M, Medial. Additional abbreviations: *pl*-VN, posterolateral vestibular nerve; bp, basilar papilla; cd, cochlear duct; HB, hindbrain. Scale bar = 16 µm in (**a**); 32 µm in (**b**,**c**); 11 µm in (**b′**,**b″**,**c′**,**c″**); 19 µm in (**d**–**f**); 8 µm in (**e′**,**f′**); 16 µm in (**f″**).

The study of type-4 chimeric embryos, which correspond to the expanded sensory areas of both the macula lagena and macula neglecta (topologically the two most posterior maculae in the chick otic placode; [5]), also displayed grafted quail neurons in both the VG and AG, with a clear heterogeneous distribution (arrows in Figure 4a–c″). In the *p*-VG, QN-positive quail neurons were detected in the most caudal half (arrows in Figure 4a), the rostral portion being almost devoid of these grafted neurons (asterisk in Figure 4a). The presence of grouped ml/mn-grafted neurons was evident in a ventral horizontal section through the *p*-VG (arrows in the *p*-VG in Figure 4b; arrows in Figure 4b″). In the most ventral aspect of the *p*-VG (Figure 4c), QN-labeled neurons were completely absent (asterisk in the *p*-VG in Figure 4c), except in the most caudal periphery (Figure 4c″; QCPN stained; see [12]). Scattered ml/mn-grafted quail neurons were also observed at the caudal level of the *a*-VG (arrows in the *a*-VG in Figure 4b; arrows in Figure 4b′). In the most ventral aspect of the *a*-VG (Figure 4c), grafted neurons were observed in the rostral areas (Figure 4c,c′), the rest of the *a*-VG did not display any QN-stained cells (asterisks in the *a*-VG in Figure 4c). The AG showed some QN-stained neurons (Figure 4d-f). The *pr*-AG was completely devoid of QN-positive cells (Figure 4d), whereas the *i*-AG and *d*-AG showed a very reduced number of grafted quail neurons in their caudal periphery (arrows in Figure 4e′,f′; see QCPN staining in Figure 4e′ for *i*-AG). These results were confirmed using QCPN immunoreactions (see Figure 4c″,e′). Figure 4h summarizes the QN/QCPN-positive grafted neuron distribution in both ganglia of the type-4 chimeric embryos (Figure 4g). 

### 3.2. Neuroblast Generation from the Cristae Presumptive Domains of the Otic Placode

**Figure 5:**
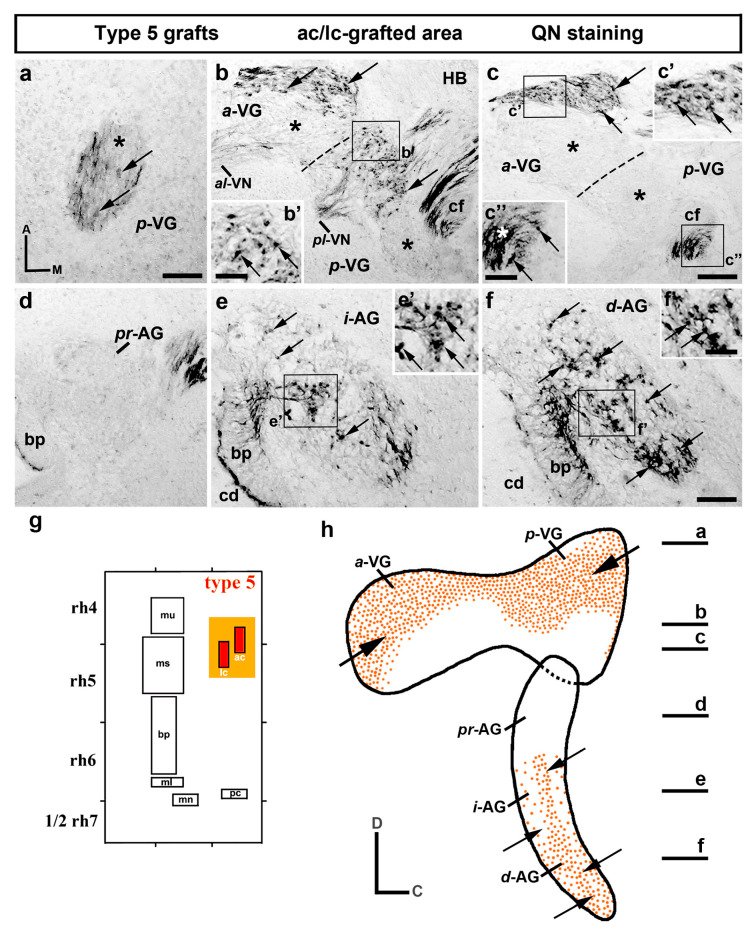
Graft-derived quail ganglionic neurons from the extended area of the anterior and lateral cristae (type-5 grafts; n = 7). (**a**–**f**) Horizontal sections through the vestibular (VG in (**a**–**c**) and acoustic (AG in (**d**–**f**)) ganglia. QN-stained grafted quail neurons occupied a large portion of both the anterior and posterior vestibular ganglion (*a*-VG and *p*-VG; arrows in (**a**–**c′**)). The asterisks in b and c indicate the areas devoid of ac/lc-grafted neurons. In the AG (**d**–**f**), the *pr*-AG did not display any quail neurons (**d**). In the *i*-AG and *d*-AG, QN-positive neurons were clearly observed (arrows in (**e**–**f′**)). (**c″**) The white asterisk indicates axons in the cochlear fascicle (cf). (**g**) Schematic representation of the type-5 grafts performed at the 10-somite stage, considering the expanded sensory area of the anterior and lateral cristae (ac and lc; dark and light orange areas). (**h**) Diagram summarizing the results, in which horizontal sections are indicated. The arrows point to the most significant accumulations of grafted quail neurons. Orientation: A, anterior; C, caudal; D, dorsal; M, Medial. Additional abbreviations: *al*-VN, anterolateral vestibular nerve; bp, basilar papilla; cd, cochlear duct; HB, hindbrain; *pl*-VN, posterolateral vestibular nerve. Scale bar = 9 µm in (**a**); 8 µm in (**b′**,**c′**); 10 µm in (**c″**); 19 µm in (**b**,**c**); 12 µm in (**d**–**f**); 6 µm in (**f′**).

The presumptive domains of the anterior and lateral cristae are located close together in the anteroventral portion of the avian otic placode [5]. Therefore, the type-5 grafts contained both sensory elements, plus small areas of their contiguous non-sensory areas (dark and light orange areas in Figure 5g). When dorsal horizontal sections through the *p*-VG of these chimeric embryos were analyzed (Figure 5a), ac/lc-grafted quail neurons were also observed (arrows in Figure 5a), with a rostral area devoid of these (asterisk in Figure 5a). In the adjacent ventral section, quail neurons were mainly present in the rostral half of the *p*-VG (arrows in the *p*-VG in Figure 5b,b′), but not in its caudal half (lower asterisk in Figure 5b). In the most ventral section across the *p*-VG (Figure 5c), graft-derived neurons were absent (lower asterisk in Figure 5c), although a few quail neurons were detected in the most caudal periphery (arrows in Figure 5c″). When the *a*-VG was analyzed (Figure 5b,c), the QN-stained neurons were grouped in the most anterior portion (arrows in the *a*-VG in Figure 5b,c,c′), while no quail-grafted neurons were detected in its caudal half (upper asterisks in Figure 5b,c). Regarding the AG, no QCPN-positive neurons were detected in the proximal portion (*pr*-AG in Figure 5d). A relevant number of quail neurons were observed in the intermediate and distal portions (*i*-AG and *d*-AG; arrows in Figure 5e,e′,f,f′). In the *i*-AG, these quail neurons were mainly concentrated in the central-medial portion (arrows in Figure 5e), whereas a more heterogenous distribution was observed in the *d*-AG (arrows in Figure 5f). The results of the type-5 experiment were confirmed using QCPN immunoreactions. Figure 5h summarizes the QN/QCPN-positive neuron distribution in both ganglia of the type-5 chimeric embryos.

**Figure 6:**
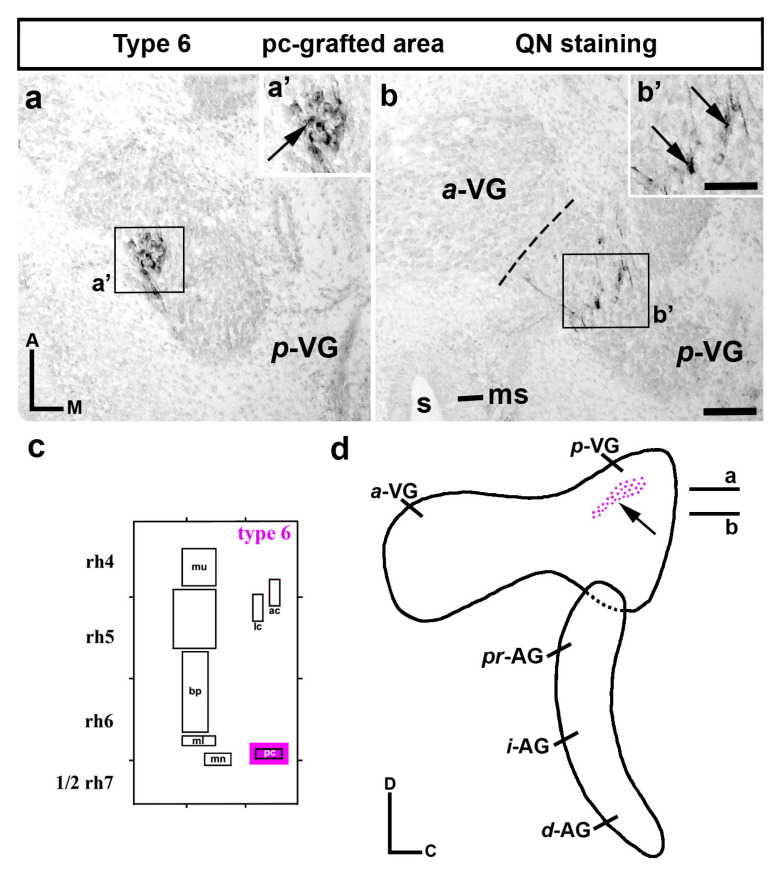
Graft-derived quail ganglionic neurons from the extended area of the posterior crista (type-6 grafts; n = 6). (**a,b**) Horizontal sections through the VG. The dotted lines in b define the separation between the anterior and posterior lobes of the VG (*a*-VG and *p*-VG). A few QN-stained grafted quail neurons were detected exclusively in the *p*-VG (arrows in (**a′**,**b′**)). The *a*-VG (**b**) and the AG were devoid of pc-grafted neurons. (**c**) Schematic representation of the type-6 grafts performed at the 10-somite stage, considering the expanded sensory area of the posterior crista (pc; purple areas). (**d**) Diagram summarizing the results, the arrow points to the restricted location of grafted neurons in the VG. Orientation: A, anterior; C, caudal; D, dorsal; M, Medial. Additional abbreviations: ms, macula sacculi; s, saccule. Scale bar = 19 µm in (**a**,**b**); 9.5 µm in (**a′**,**b′**).

Finally, the type-6 chimaeric embryos, containing the presumptive area of the posterior crista and its associated non-sensory areas, were also analyzed (Figure 6; Sánchez-Guardado et al., 2014). A few pc-grafted neurons were observed in the *p*-VG (arrows in Figure 6a′,b′), with the *a*-VG and the entire AG being completely devoid of QN-stained cells (Figure 6b for *a*-VG). Figure 6d summarizes the distribution of QN/QCPN-positive grafted neurons in the type-6 experimental embryos.

## 4. Discussion

### 4.1. The Pro-Sensory Domain of the Otic Placode

The specification of different cell types that make up the membranous labyrinth occurs through a series of cell fate decisions, which take place in specialized areas and in a very orderly fashion. Questions about fate assignment in the incipient otic placode and further clonal expansion require the determination of the origin of early populations of epithelial-derived components. In avians, an exhaustive fate map study of the otic placode, 10-somite stage, using the chick–quail experimental approach as an efficient labelling method, accurately determined the presumptive territories of each component of the avian inner ear [5]. The existence of three dorsoventrally arranged anteroposterior domains was clearly demonstrated, from which specific sensory and non-sensory elements develop. Focusing on the presumptive domains of sensory elements, all maculae and the macular-derived basilar papilla are generated from the intermediate domain, whereas all cristae arise from the ventralmost domain [5]. This topologic specification in the otic placode epithelium seems to be organized by diffusible molecules released from adjacent tissues and the otic epithelium itself, governing the activation and repression of key positional genes in pattern-generating systems [3,6,16,18,19,24,28,81]. Following the above-mentioned fate map study, recent works have shown that, at stage HH10, all small grafted areas, comprising individual or close sensory elements plus a small portion of their contiguous non-sensory epithelium (epithelia), contain precursors of otic neuroblasts. Moreover, grafted ganglion neurons do not derive from the same sites of the otic placode, they innervate later when development proceeds [48,82]. 

Specification of the otic neuroblast lineage is the first cell differentiation event in the inner ear development, being simultaneous with or immediately following the determination of the incipient otic pacode [22,23,62]. Although important progress has been made in recent decades, some cellular and molecular events controlling when, where, and how acoustic and vestibular neural fates are specifically acquired remain unknown in detail [24,28,83,84]. In the chick otic placode, the asymmetric expression of several markers has been identified, with these patterns reflecting the compartmentalization of the otic placode. An anterior-ventral to posterior-dorsal boundary could be defined at an angle of about 45° to the anterior-to-posterior axis of the embryo [33]. The most anterior domain of the otic placode corresponds to the *Fgf10*-expressing domain, known as the proneural-sensory domain, from which all sensory elements and otic neurons are generated [24]. Together with the expression of the *Fgf10* gene, the expression of the *Neurogenin1*, *Delta1*, *Hes5*, and *Sox3* genes are also restricted to the anterior neurogenic domain, whereas the *Lmx1b* and *Tbx1* expressions are limited in the posterior non-neurogenic domain in a complementary manner [24,83,84]. Diffusible signals from the adjacent tissue, in particular from the ectoderm, play a key role in the specification of neurogenic versus non-neurogenic territories in the early chick otic placode [19,20,30]. Thus, the expressions of *Sox3* and *Lmx1b* genes are regulated by *FGF8* and *BMP*, respectively, with *FGF8* from the most anterior portion of the otic placode acting as the main signal restricting the otic neurogenic domain [84,85]. In this molecular context, the final determination of these two complementary domains along the anterior-to-posterior axis of the chick embryo could take place after otic placode formation [86].

### 4.2. Segregation of Acoustic and Vestibular Neuronal Precursors by Otic Vesicle Stage

Although many molecular and cellular aspects of the AG and VG formation have been well described, the intricate network of signaling pathways and transcription factors that underlie their genesis as independent neuronal structures and how they are precisely segregated along the axes of the developing otic anlagen are only now being understood. Birth-dating studies have shown that vestibular neurons seem to be postmitotic before cochlear neurons [87]. Thus, differences in the timing of the birth of vestibular and acoustic neurons may also reflect differences in the origin of these two neuronal populations. It has also been suggested that neuroblasts constituting the developing AG and VG could be specified before they leave the otic epithelium, in two contiguous domains within the ventral wall of the otic vesicle. These two areas are separated by a medio-lateral boundary and associated with the utricular and saccular maculae, respectively [34,41]. The VG and the utricular macula are specified before the AG and saccular macula at the otic cup stage [41]. The subdivision of the neurogenic domain into vestibular and auditory regions was also suggested when analyzing the expression of relevant neural markers. In the developing acoustic-vestibular ganglion, NeuroM-positive neurons were detected in the distal-most domain [24], precisely where the vestibular neurons are placed [21,23], the *NeuroM* gene being thus an excellent candidate for conferring vestibular identity. On the other hand, *GATA-3* may be directly involved in auditory neuron specification [49,50,51,52,88,89]. In mice *GATA-3*-labelling cells were detected in the differentiating spiral ganglion neurons, but not in the vestibular ganglion [49,50,51,52]. In addition, *GATA-3* null mice displayed a severe reduction of cochlear sensory neurons [50]. Although several studies have reported that a subset of vestibular neurons could express *GATA-3* at some stage of embryonic development [88,90], *GATA-3* is generally associated with auditory neuron development [49,50,51,52,89]. Although *NeuroD* is also a key factor in early cell fate determination and later delamination of otic neurons [13,24,40,91,92], vestibular neurons express *NeuroD*, but not *GATA-3*, in the E13.5 mammalian inner ear [52]. 

It is well known that otic neuroblast differentiation is governed by another bHLH transcription factor, *Neurogenin1*, acting as a positive upstream regulator in the *NeuroD* signaling cascade [93]. Thus, null *ngn1* mutants display an absence of both auditory and vestibular neurons [93]. Using the *Ngn1-CreER^T2^* transgenic mouse line, it was reported that, although neurons of the vestibular and auditory ganglia arise from a common neurogenic domain, they are generated from independent populations of *Ngn1*-expressing precursors during two overlapping waves of otic neurogenesis during the otic vesicle stage. The earliest *Ngn1*-expressing cells are almost exclusively vestibular neurons, whereas the late *Ngn1*-positive cells are cochlear neurons [53]. In this sense, it is worth remarking that an exhaustive study of the gene expression patterns in developing auditory neurons from the E12 to E15 stages in mouse embryos identified cell type-specific molecular factors differentially enriched in the acoustic versus vestibular neurons at mid and late developmental stages, with this dataset being useful for functional studies [90]. 

### 4.3. No Segregation of Acoustic and Vestibular Neuronal Precursors at the Otic Placode Stage

Using the lipophilic dyes DiI and DiO at the HH10-HH17 stages, mostly to label the proneural domain in the anterior-medial(dorsal) domain of the otic placode/cup and for analyzing the experimental embryos at stage HH30–32, it was concluded that the precursors for vestibular and auditory neurons are segregated early in the otic placode/cup epithelium, rarely mixing together [54]. Thus, vestibular and auditory neuroblasts originate separately from the anterior-lateral(ventral) and posterior-medial(dorsal) portions of the otic cup, respectively. This fate mapping study showed that: (1) the antero-lateral region of the proneural domain mainly generates neuroblasts that innervate the anterior and lateral cristae; (2) the antero-medial region produces neurons that project into the posterior crista and the maculae; and (3) the posteromedial domain mainly generates neurons that innervate the basilar papilla. However, the macula lagena and macula neglecta were not considered. Our fate mapping study also showed that otic neuroblasts presumably always originate in very close proximity to the sensory patches that they will later innervate. Therefore, stereotyped connections depend on the areas in which the neurons are born [54]. 

**Figure 7:**
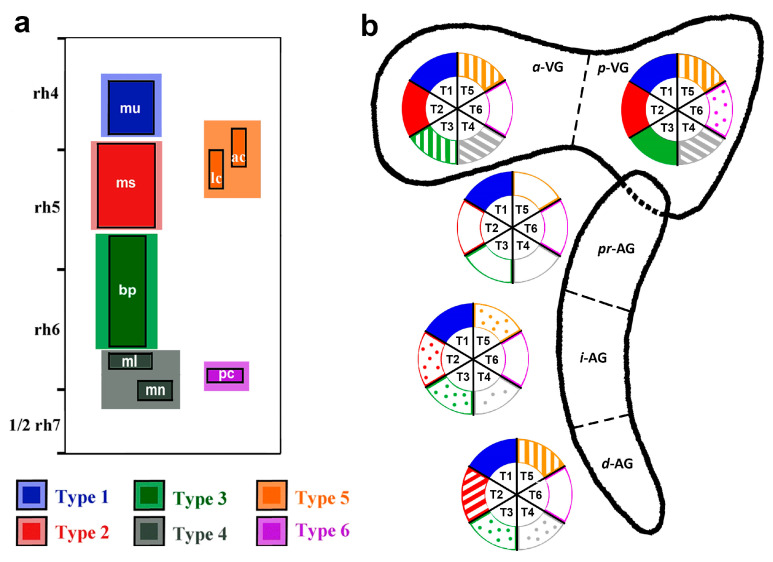
Avian otic placode schematic representation at stage HH10 (10 somites), showing the grafted ectoderm portions of each transplant type. (**b**) Schematic representation displaying the neuronal contribution of each graft type to the different acoustic–vestibular ganglion portions (a-VG, p-VG, pr-AG, i-AG, and d-AG). Each graft type is represented by the same color code used in (**a**). The neuronal contribution of each graft is denoted as high (full colored), medium (stripes), low (dots), or no contribution (white).

Using QN immunoreactions, which allow us to visualize the axons of quail neurons in chimeric embryos, the sensory connection patterns generated by diverse graft-derived neuronal populations were determined [48]. All small grafts of the otic placode, at the 10-somite stage, containing the presumptive territory of one or two sensory elements plus a small contiguous portion of associated non-sensory elements, generate otic neuroblasts [48]. Thus, the present work confirmed that all experimental grafts, types 1–5, contributed with quail neurons to both VG and AG (Figure 7). The area of the otic placode associated with the posterior crista (type 6), located in the most caudal-ventral portion, participated with very few neuroblasts in the VG and with none in the AG. According to the fate map referred to above, the type-6 transplants took a placodal portion, mainly in the *Lmx1*-positive (non-neurogenic) and *Fgf10*-negative (neurogenic) domain [24,32,48]. Thus, the type-6 transplants contained a much-reduced portion of the neurogenic territory, so that the transplanted region generated significantly fewer otic neuroblasts. The type-1–5 transplants, clearly included in the neurogenetic domain of the otic placode, generated neuroblasts that were integrated in both VG and AG, indicating that there would be no segregation of vestibular or acoustic neuroblasts in the incipient otic placode. Considering the VG, the type-1–5 transplants generated neurons that were located in the anterior and posterior lobes. These neurons occupied continuous portions from their anterior to posterior parts. The absence of QN/QCPN-positive cell patches observed in the type-1 and -2 grafts, marked with asterisks in their respective figures, strongly suggests that, although there is no segregation of vestibular or acoustic neuroblasts in the avian otic placode, subpopulations of, at least, vestibular neuroblasts with a specific origin should be considered. More detailed studies should be carried out using innovative cell lineage approaches.

The analysis of the distribution of graft-derived neurons in the AG showed that all graft types, with the exception of type 6, contributed neurons to this ganglionic structure (Figure 7). It should be noted that the most anterior-dorsal territories of the otic placode, associated with the utricular macula (type 1), apparently provided a higher number of auditory neurons, when compared with the rest of the graft types. The quail neurons from this type of experiment invaded the entire AG from the base to the apex and were included within the three proximal-to-distal anatomical subdivisions (*pr*-AG, *i*-AG, and *d*-AG). The grafted placodal presumptive domains of the saccular macula (type 2), basilar papilla (type-3 grafts), macula lagena and macula neglecta (type-4 grafts), and anterior and lateral cristae (type-5 grafts) also participated in the AG formation, although to a lesser extent. It is worth to remarking that the *pr*-AG was devoid of QCPN- and QN-positive neurons in the type-2–5 experiments. This absence of neurons in the *pr*-AG could be due to the apex–base gradient of the innervation pattern of the developing auditory sensory element and the tonotopic projections of these neurons into the cochlear nuclei in the hindbrain [8,18,46,94,95,96]. The existence of subpopulations of auditory neuroblasts with different origins in the early otic placode could also be considered. Furthermore, transplants from the presumptive territory of the basilar papilla not only produced auditory neurons but also vestibular neurons, as was shown in preliminary works [48].

## 5. Conclusions

In the developing avian inner ear, there is no segregation of acoustic and vestibular neuronal precursors at the otic placode stage. The specification of otic neuroblasts is an early event that takes place during the invagination of the otic placodes, with progressive step-by-step changes in cellular and molecular aspects as the development progresses. Despite the numerous studies carried out in recent years, the molecular and cellular mechanisms governing how the VG versus AG neuroblasts are originated remains to be determined to a large extent. Further descriptive and experimental studies are necessary to better characterize the gene expression mosaic of transcription factors and signaling pathways governing the neuronal fate acquisition in the incipient otic placode and the consequent gradual restriction of neuroblast origins that takes place as development proceeds. Cell lineage studies with more precise methodologies should be carried out.

## Data Availability

Data are contained within the article, and materials can be requested from the authors upon reasonable request.

## Supplementary Materials

The following supporting information can be downloaded at: https://www.mdpi.com/article/10.3390/biology12030453/s1, Figure S1: Anatomical characteristics of the acoustic and vestibular ganglia at stage HH34. Horizontal sections from the dorsal (**a**) to ventral (**i**) aspects of the acoustic and vestibular ganglia treated with HuC/D immunoreactions. The vestibular ganglion shows anterior (*a-*VG) and posterior (*p*-VG) portions, fused in the central sections (**b**–**e**), but not in the most dorsal (**a**) and ventral (**f**) sections. (**b**–**e**) The dotted lines define the separation between the anterior and posterior lobes of the VG (*a*-VG and *p*-VG). Along the proximal-to-distal axis of the cochlear duct (cd in (**e**–**i**)), the acoustic ganglion can be subdivided in three portions: proximal (*pr*-AG in (**e**–**g**)), intermediate (*i*-AG in (**h**)), and distal (*d*-AG in (**i**)). Orientation: A, anterior; M, Medial. Additional abbreviations: HB, hindbrain; s, saccule. Scale bar = 20 µm in (**a**–**f**); 14 µm in (**g**–**i**). Figure S2: Schematic representation showing the nerves that innervate the different sensory elements arising from the vestibular ganglion (VG), as well as axons arising from the auditory ganglion (AG). The anterior vestibular nerve (*a*-VN) innervates the anterior and lateral cristae, while the anterior-lateral nerve (*al*-VN) innervates the macula utriculi. The posterior-lateral vestibular nerve (*pl*-VN) innervates the macula sacculi, while posterior nerve (*p*-VN) innervates the posterior crista and the macula neglecta.

## Abreviations

ac	anterior crista
AG	acoustic ganglion
AVG	acoustic-vestibular ganglion
*a*-VG	anterior vestibular ganglion
*a*-VN	anterior vestibular nerve
*al*-VN	anterior-lateral vestibular nerve
bp	basilar papilla
cd	cochlear duct
cf	cochlea nerve fascicle
*d*-AG	distal acoustic ganglion
HB	hindbrain
*i*-AG	intermediate acoustic ganglion
lc	lateral crista
ml	macula lagena
mn	macula neglecta
ms	macula sacculi
mu	macula utriculi
pc	posterior crista
*p*-VG	posterior vestibular ganglion
*p*-VN	posterior vestibular nerve
*pl*-VN	posterior-lateral vestibular nerve
*pr*-AG	proximal acoustic ganglion
