# Supplementary material for: Origin of Neuroblasts in the Avian Otic Placode and Their Distributions in the Acoustic and Vestibular Ganglia
Source: Biology (Basel). 2023 Mar 15;12(3):453. doi: 10.3390/biology12030453 (PMC10045822; doi:10.3390/biology12030453)
Supplement: Supplementary file 1 [file biology-12-00453-s001.zip › Supplementary Material.pdf]

Supplementary Material

Figure S1

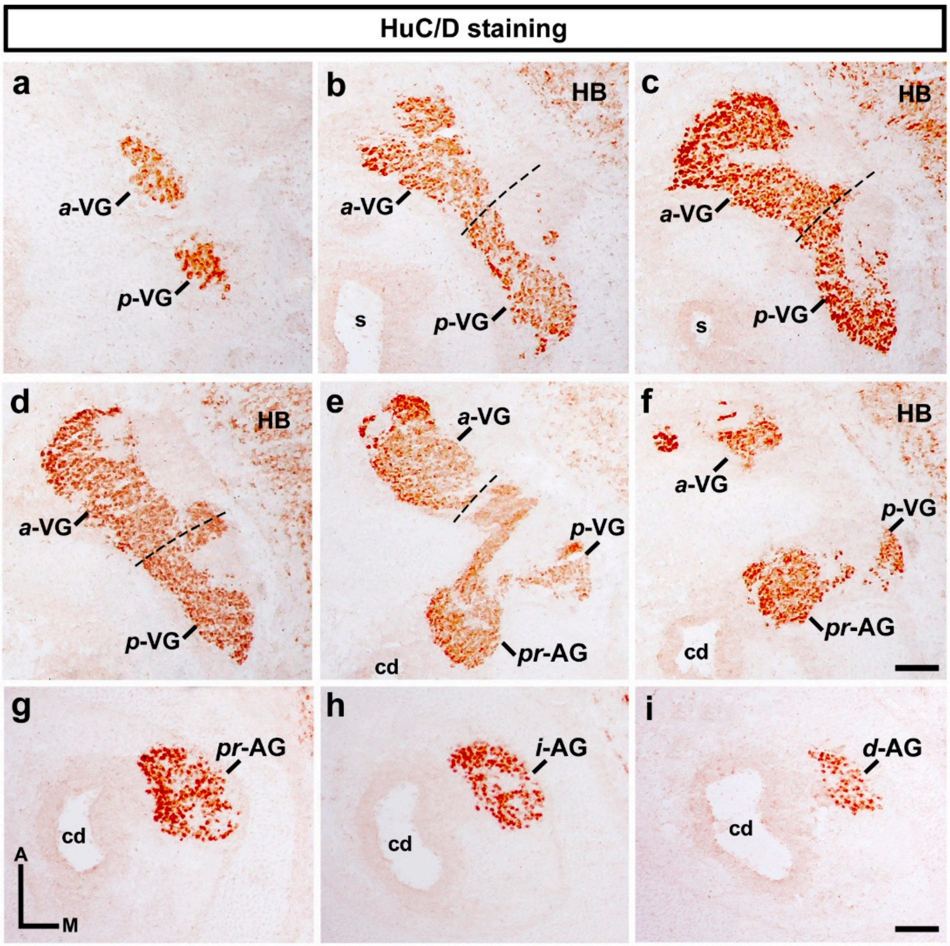

Suppl. Figure 1

Figure S1: Anatomical characteristics of the acoustic and vestibular ganglia at stage HH34. Horizontal sections from the dorsal (**a**) to ventral (**i**) aspects of the acoustic and vestibular ganglia treated with HuC/D immunoreactions. The vestibular ganglion shows anterior (*a*-VG) and posterior (*p*-VG) portions, fused in central sections (**b**-**e**), but not in the most dorsal (**a**) and ventral (**f**) sections. (**b-e**) The dotted lines define the separation between the anterior and posterior lobes of the VG (*a*-VG and *p*-VG). Along the proximal-to-distal axis of the cochlear duct (cd in (**e-i**)), the acoustic ganglion could be subdivided in three portions: proximal (*pr*-AG in (**e**-**g**)), intermediate (*i*-AG in (**h**)), and distal (*d*-AG in (**i**)). Orientation: A, anterior; M, Medial. Additional abbreviations: HB, hindbrain; s, saccule. Scale bar = 20 μm in **f** (**a-f**); 14 μm in **i** (**g-i**).

Figure S2

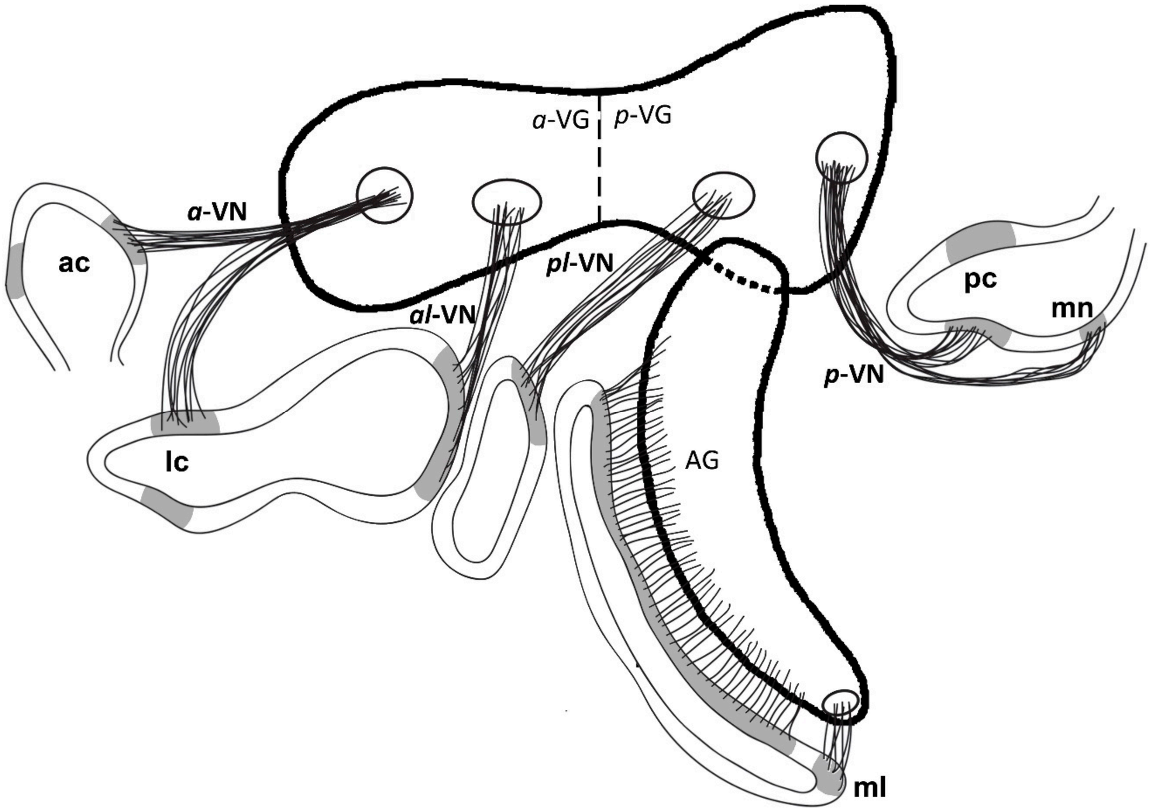

Figure S2: Schematic representation showing the nerves that innervate the different sensory elements arising from the vestibular ganglion (VG), as well as axons arising from the auditory ganglion (AG). The anterior vestibular nerve (*a*-VN) innervates the anterior and lateral cristae, while the anterior-lateral nerve (*al*-VN) innervate the macula utriculi. The posterior-lateral vestibular nerve (*pl*-VN) innervate the macula sacculi, while posterior nerve (*p*-VN) innervates the posterior crista and the macula neglecta.
